# Mokken scale analysis of the Internet Gaming Disorder Scale–Short-Form and the Gaming Disorder Test

**DOI:** 10.1016/j.abrep.2024.100567

**Published:** 2024-10-28

**Authors:** Laura Maldonado-Murciano, Halley M. Pontes, Maite Barrios, Juana Gómez-Benito, Georgina Guilera

**Affiliations:** a Faculty of Psychology, University of Barcelona, Spain; b Institute of Neurosciences, University of Barcelona, Spain; c Department of Psychology, International University of Catalonia, Spain; d School of Psychological Sciences, Birkbeck, University of London, United Kingdom

**Keywords:** IGDS9-SF, GDT, Mokken Scale Analysis, Item discrimination, Item difficulty, Gaming Disorder, Psychometrics

## Abstract

In recent years, research on disordered gaming has grown substantially with researchers developing different psychometric tools for assessing it. Two of the most prominent assessment tools are the Internet Gaming Disorder Scale–Short-Form (IGDS9-SF) and the Gaming Disorder Test (GDT), which evaluate disordered gaming under the American Psychiatric Association (APA) and the World Health Organization (WHO) frameworks, respectively. The main aim of this study was to assess and compare the scalability, reliability, and validity of both scales to determine if they effectively assess disordered gaming in a normative sample, through the Mokken Scale Analysis (MSA). A sample of 605 participants (42.31 % female, mean_age_ = 23.98 years, *SD* = 9.21 years) was recruited for the present study. Results showed that both the IGDS9-SF and GDT were unidimensional, with all items presenting latent monotonicity fitting in the Monotone Homogeneity Model (MHM). Item characteristic curves did not intersect and presented with adequate fit in the Double Monotonicity Model (DMM). These findings further support the psychometric adequacy of the IGDS9-SF and GDT, attesting to their suitability to assess disordered gaming.

## 1 Introduction

Scientific interest in disordered gaming and its effects grew substantially over the last decade (Pontes & Griffiths, 2020), leading to an increase in the global scientific production in behavioural addiction research, including disordered gaming (Sixto-Costoya et al., 2021). The inclusion of ‘Internet Gaming Disorder’ (IGD) as a tentative disorder in Section 3 of the 5^th^ revision of the *Diagnostic and Statistical Manual of Mental Disorders* (DSM-5; APA 2013) by the American Psychiatric Association (APA) and the formal recognition of ‘Gaming Disorder’ (GD) as an official mental health disorder by the World Health Organization (WHO) in the 11th version of the *International Classification of Diseases* (ICD-11; WHO, 2022) have likely contributed to the broader scientific interest in disordered gaming.

Disordered gaming is currently conceptualised under both the APA and WHO frameworks (Montag et al., 2021, Pontes et al., 2022, Zhou et al., 2020). Within the APA framework, IGD is defined as ‘persistent and recurrent use of the internet to engage in games, often with other players, leading to clinically significant impairment or distress’ (APA, 2013, p. 795). Moreover, under the WHO framework, GD is understood as ‘an excessive pattern of gaming behavior (online and/or offline) reflected by lack of control, greater priority given to gaming over other life interests and activities, and continuation of the behavior despite adverse outcomes’ (WHO, 2022, Section 6C51).

Since its definition, disordered gaming has been found to have a prevalence of 3.3 % in the global population, according to a recent meta-analysis (Kim et al., 2022). It significantly impacts quality of life (Phan et al., 2019) and has notable comorbidity with other disorders such as depression (Ostinelli et al., 2021) and other behavioral addictions (Pontes, 2017, Rozgonjuk et al., 2023). In terms of health-related outcomes, the meta-analysis by Männikkö et al. (2020) showed that disordered gaming is linked to depression, anxiety, obsessive–compulsive disorder, and somatization. Several important risk factors have been identified by Gao et al. (2022) in their *meta*-analysis, including, but not limited to, stress, higher time spent gaming, family disfunction, interpersonal problems, emotional distress, low self-esteem, and existing mental disorders (e.g., hyperactivity/inattention, anxiety, and depression).

Due to public health relevance, researchers have recently developed standardized psychometric tools to assess disordered gaming under the APA and WHO diagnostic frameworks (see King et al., 2020). Two of the most commonly used tools are the Internet Gaming Disorder Scale–Short-Form (IGDS9-SF; Pontes & Griffiths, 2015) and the Gaming Disorder Test (GDT; Pontes et al., 2021), which assess disordered gaming under the APA and the WHO framework, respectively. Despite the relatively large body of evidence supporting the sound psychometric properties of these two tools (Evren et al., 2020, Gomez et al., 2019, Maldonado-Murciano et al., 2020, Maldonado-Murciano et al., 2021, Pontes et al., 2021, Pontes and Griffiths, 2014, Pontes and Griffiths, 2015, Schivinski et al., 2018), no previous research has yet investigated whether there is a stable hierarchy in terms of item difficulty within the two scales across different participants, nor whether the sum score of each item can be used to order respondents on the latent trait being measured (i.e., disordered gaming).

The use of Mokken Scale Analysis (MSA) is suitable for investigating the relationship between items and latent traits and has evolved from the Guttman method of examining hierarchies of items within a scale (Watson et al., 2012). The MSA is a type of Non-parametric Item Response Theory (NIRT) model, a more flexible model which present fewer restrictions on the data compared to parametric IRT models (such as Rasch models). This flexibility enhances the range of items for which behavior can be accurately analyzed and accurately explained in the analysis (Meijer et al., 1990). In NIRT models, if the model fits the data, the sum score of each participant can be used to order respondents based on the severity of the latent trait measured (see Koopman et al., 2022; and van der Ark & Bergsma, 2010).

The literature supports the use of MSA, as this method has been shown to be useful in assessing the item hierarchy in several scales (Abdelhamid et al., 2020, Friedrich et al., 2015, Muncer and Speak, 2016). MSA explores whether items of a scale are hierarchically ordered by degree of difficulty and detects any violations (e.g., test-takers agreeing with a ‘difficult’ item while not agreeing with an ‘easier’ one). Moreover, the scalability coefficients allow the assessment ofitem quality within a given set of items or, when creating a new tool, to use them as an item selection procedure (Sijtsma & Van der Ark, 2017). In practical terms, MSA allows the evaluation of the degree to which individuals can be meaningfully ordered on the construct such that individuals with higher scores exhibit more symptoms. Likewise, MSA enables the assessing the degree to which symptoms have a consistent interpretation between participants, such that the relative order of symptoms is the same for those with relatively low or high levels of addiction (Sijtsma et al., 2011).

As for the construct of disordered gaming, to the best of our knowledge, only one study has been conducted using MSA (Finserås et al., 2019). Nevertheless, the present study extends the findings reported by Finserås et al. (2019) by addressing two main points. Firstly, this study will focus on the two most prominent scales for disordered gaming (i.e., IGDS9-SF and GDT) developed under the current APA and WHO diagnostic frameworks. Secondly, this study will provide findings not tied to a specific developmental stage as the IGDS9-SF and GDT evaluate disordered gaming more broadly across the lifespan and not only on adolescents.

The primary objective of this study is to evaluate and compare the psychometric properties of the IGDS9-SF and GDT in measuring disordered gaming based on different diagnostic frameworks. Specifically, this study aims to assess and compare the scalability, reliability, and validity of these two instruments to determine how and whether they effectively capture disordered gaming in a normative sample of adults. By applying MSA (see Mokken, 1971; and Mokken & Lewis, 1982) to both instruments, the study seeks to identify the extent to which the items of each instrument, as well as the instruments themselves, are effective for use in psychological research and clinical assessment practice. The results of this comparative psychometric study will help researchers and health practitioners in the selection of the most adequate tool for measuring disordered gaming, ensuring reliable and valid assessments that can inform effective interventions and research outcomes.

## 2 Methods and materials

### 2.1 Participants and procedures

Similar to other validation studies of the IGDS9-SF and GDT (e.g., Beranuy et al., 2020, Maldonado-Murciano et al., 2020) the target population for this study was Spanish-speaking individuals aged 16 or older who played video games at least once a week. Participants were recruited through an online survey, which was advertised via multiple social media platforms (i.e., Facebook, Instagram, Reddit, and Twitter) and the online course management system of a second-year course in the Psychology degree program at the University of Barcelona. All study procedures were conducted in accordance with the Declaration of Helsinki and approved by the Bioethics Committee of the University of Barcelona. All participants provided their consent online. In accordance with Spanish legislation (i.e., Article 7.1 of the Spanish Law 3/2018 of December 5, on the Protection of Personal Data and Guarantee of Digital Rights, 2018), parental consent was not required for minors, as for this type of study, minors can provide their own consent starting at the age of 14.

A sample of 1,023 participants was initially recruited for the study. However, a total of 418 participants were removed due to the following reasons: not having played any video games in the last 12 months (*n* = 297, 29.03 %), playing more hours a week than hours in the week (*n* = 79, 7.72 %), reporting not having played video games during the entire week (0 hour per week, *n* = 11, 1.07 %), and not completing the whole survey (*n* = 31, 3.03 %). As a result, a final sample of 605 participants was deemed eligible and used in the statistical analyses (available on request), with 23.97 % of the participants recruited through the psychology course and 76.03 % through the advertisements. In terms of sample characteristics, 256 participants were female (42.31 %), and the mean age was 23.98 years (*SD* = 9.21 years; range: 16–73 years). Regarding educational level, 57.85 % of the participants had completed secondary studies, followed by 28.92 % who had achieved higher education. Additionally, 11.74 % had primary education, and the remaining 1.49 % did not have any formal education (see Table 1 for detailed sociodemographic and gaming behaviour characteristics).

**Table 1:** Descriptive sociodemographic and gaming behaviour data from the sample (*n* = 650).

**Variables**	**Mean**	**SD**	**Min.**	**Max.**
**Age**	23.98	9.21	16	73
**Hours playing** video **games on working days**	1.92	2.07	0	16
**Hours playing** video **games on free days**	3.39	2.85	0	24
**Max. hours ever played**	**39.86**	**28.65**	**1**	**89**

**Note:** SD = standard deviation; Min. = minimum value; Max. = maximum value,

### 2.2 Measures

Sociodemographic and gaming behavior data: The survey collected sociodemographic and gaming behavior data aligned with previous similar psychometric studies (e.g., Maldonado-Murciano et al., 2020, Maldonado-Murciano et al., 2021), including participants’ gender, age, weekly time spent gaming, and maximum hours ever played in a row. It is important to note that the sociodemographic information collected differed slightly between the social media and the course questionnaires (e.g., birthplace was only asked of participants recruited through social media).

Internet Gaming Disorder Scale–Short-Form (IGDS9-SF; Pontes & Griffiths, 2015): The IGDS9-SF is a nine-item standardized test designed to assess disordered gaming under the nine criteria of the APA framework: (i) *preoccupation;* (ii) *withdrawal*; (iii) *tolerance*; (iv) *loss of control*; (v) *loss of interests*; (vi) *continuation*; (vii) *deception*; (viii) *escape*; and (ix) *negative consequences*. The items are rated using a five-point response scale ranging from 1 (never) to 5 (very often), with higher scores indicating greater levels of disordered gaming symptoms. The Spanish version of the IGDS9-SF used in this study has shown excellent psychometric properties (Beranuy et al., 2020, Maldonado-Murciano et al., 2020, Sánchez-Iglesias et al., 2020). In the present study, the IGDS9-SF scores showed high internal consistency (α = .84, ω = .86).

Gaming Disorder Test (GDT; Pontes et al., 2021): The GDT is a brief, four-item psychological test developed to assess disordered gaming under the WHO framework. All items are rated on a five-point scale ranging from 1 (never) to 5 (very often), yielding total scores ranging from 4 to 20 points, with higher scores indicating a greater degree of disordered gaming symptoms. The first three items of the GDT reflect (1) *impaired control over gaming,* (2) *increased priority given to gaming*, and (3) *continuation despite negative consequences*, while the fourth item assesses potential functional impairments by evaluating gamers’ (4) *experience of significant problems in life* due to GD. For this study, the Spanish version of the GDT was used (Maldonado-Murciano et al., 2021) as it has been shown to exhibit adequate psychometric properties. In the present study, the GDT scores showed high internal consistency (α = .81, ω = .83).

### 2.3 Statistical analysis

The NIRT models typically assessed with MSA are the Monotone Homogeneity Model (MHM), which implies ordering individuals on a scale based on their total scores, and the Double Monotonicity Model (DMM), which implies ordering both persons (as in the MHM) and items in terms of difficulty, and tests whether this item ordering is invariant across the latent variable continuum (Mokken & Lewis, 1982). As originally proposed by Mokken and Lewis (1982), the MHM is sufficient for test construction. However, if the test is to be administered, the DMM is also necessary to ensure that the order of item difficulties remains consistent for all participants. This stability can be studied by comparing the relative item ordering between participants with different overall levels on the construct. The present study assessed whether the IGDS9-SF and GDT fit both models. As these scales include polytomous items, the MSA for polytomous scales proposed by Molenaar, 1991, Molenaar, 1997 was adopted.

Four assumptions are used to define the most popular NIRT models: unidimensionality, local independence, latent monotonicity, and no intersection of Item Response Functions (IRFs), with the latter being only used in the case of the DMM (van der Ark, 2012). Unidimensionality signifies that all the items in the scale measure a single latent variable (Sijtsma & Van der Ark, 2017). Local independence implies that the responses of the items are unrelated between them when the level of the latent variable is controlled for (van der Ark, 2012). Latent monotonicity refers to the increasing probability of a higher item score as the level of the latent trait increases (Watson et al., 2012). Finally, non-intersection assumes that IRFs do not intersect (Watson et al., 2012), indicating that items are ordered by difficulty.

To explore whether the MHM and DMM models fit the IGDS9-SF and GDT items, various analyses were conducted. Two Confirmatory Factor Analyses (CFA) were fitted to assess the unidimensionality of the IGDS9-SF and GDT (Smits et al., 2012). Given that the items on both scales are on a Likert-point scale, models were estimated using the Weighted Least Aquares Mean and Variance adjusted (WLSMV) estimator. The fit of the models was evaluated using the following indices: Tucker-Lewis Index (TLI), Comparative Fit Index (CFI), and Standardized Root Mean Square Residual (SRMR). The suggested guidelines of Hu and Bentler (1999) were followed when interpreting the goodness of fit, with values of CFI ≥ 0.95, TLI ≥ 0.95, and SRMR ≤ 0.08 considered satisfactory fit. The Item Step Response Functions (ISRF) and Item Response Functions (IRF) were obtained to observe the monocity of the items individually.

Three scalability coefficients (van der Ark, 2012), based on Loevinger’s coefficient *H* of homogeneity, were obtained: the item scalability coefficient (*H_j_*), the item pairs scalability coefficient (*H_jk_*), and the scale total scalability coefficient (*H*). When a scale fits the MHM, the *H_j_* can be interpreted as an index of item ‘discrimination’, that is, how well the item can distinguish between respondents with low and high scores on the latent trait (McGrory et al., 2014), with higher *H_j_* values indicating greater discrimination (Sijtsma et al., 2011). Similarly, coefficient *H* is a weighted mean of the item coefficients and can be interpreted as an average discrimination power, indicating the precision of ordering persons by their total score on the latent trait (Sijtsma et al., 2011).

For a set of items to form a Mokken scale, two necessary conditions must be met: all values of *H_jk_* must be greater than 0, and *H_jk_* must be greater than an a priori chosen criterion *c* (Watson et al., 2012). In this study, the recommendation from previous studies specifying *c* = 0.30 was adopted (Mokken, 1971, Mokken and Lewis, 1982). Moreover, all values of *H* need to exceed 0.30 to show evidence of an adequate fit of the item in the scale. If *H* is between 0.30 and 0.40, it is considered a weak scale, medium scale if it is between 0.40 and 0.50, and strong scale if it is above 0.50 (Mokken & Lewis, 1982).

Furthermore, the Automated Item Selection Procedure (AISP) was applied to identify items that satisfy the Mokken scale conditions. The AISP is a sequential method that assesses whether all items fit within the scale by evaluating their scalability indices, aiming to achieve the maximum total *H* (Smits et al., 2012). In the present study, four different lower bounds (*c*) were tested as recommended in the literature (Mokken, 1971): 0.05, 0.10, 0.30, and 0.50. For each analysis, the inter-item covariances need to be positive and exceed these lower bounds to confirm the unidimensionality of the items ($H_{j}\geq c\geq 0$). However, while MSA and the AISP procedure can provide evidence of unidimensionality, they are not definitive tests, and additional analyses (e.g., CFA) are often required to comprehensively test unidimensionality.

The Manifest Item Invariant Ordering (MIIO; Ligtvoet et al., 2010) was also analysed to test whether items can be ordered in terms of difficulty, forming a hierarchical structure, and whether this ordering is consistent across the latent trait. The method involves evaluating the IRFs for each pair of items to ensure that they do not intersect (van der Ark, 2012). In cases where item pairs do not hold MIIO (i.e., violations of the MIIO), they need to be removed from the set of items, and the scalability coefficient *H^T^* must be assessed with the remaining items, as it expresses the degree to which the order of the items remains invariant (Sijtsma & Van der Ark, 2017). The *H^T^* coefficient was interpreted using the established guidelines proposed by Mokken (1971): weak ordering (0.3 < *H^T^ ≤* 0.4), moderate ordering (0.4 < *H^T^ ≤* 0.5), and strong ordering (*H^T^* > 0.5).

Finally, the reliability of the IGDS9-SF and GDT was assessed using Cronbach’s alpha, the lambda-2 statistic (λ^2^) (Guttman, 1945), the Molenar-Sijtsma statistic (MS) (Sijtsma & Molenaar, 2002), which has a more negligible bias for MSA, and the Latent Class Reliability Coefficient (LCRC), an unbiased statistic of test-score reliability (van der Ark et al., 2011).

Statistical analyses were performed using R version 4.1.2 (R Core Team, 2022) and the following packages: *dplyr* V 1.0.7 (Wickham et al., 2018), *psych* V 2.1.9 (Revelle, 2015), and *Mokken* V 3.0.6 (van der Ark et al., 2021).

## 3 Results

The fit indices of the two CFAs confirmed the unidimensionality of both the IGDS9-SF (CFI = 1.0, TLI = 1.0, SRMR = 0.02) and the GDT (CFI = 1.0, TLI = 1.0, SRMR = 0.05), suggesting in turn that the assumption of local independence is met (Sijtsma et al., 2011). The resulting path diagrams are shown in Figure S1 of the Supplementary Material.

Figures S2 and S3 of the Supplementary Material present the ISRFs and IRFs for the IGDS9-SF and the GDT, respectively, while Table 2 shows the results of the MSA for the items of the scales.

**Table 2:** Descriptive statistics and Mokken Scale Analysis results of the IGDS9-SF and GDT (*n* = 650).

Scale/Items	Mean	SD	H_j_	SE	AISP				v. MIIO
					0.05	0.10	0.30	0.55	
IGDS9-SF									
Item 1: *preoccupation*	1.96	1.09	0.48*	0.03	1	1	1	1	0
Item 2: *withdrawal*	1.52	0.79	0.51**	0.03	1	1	1	1	0
Item 3: *tolerance*	1.54	0.87	0.48*	0.03	1	1	1	1	0
Item 4: *loss of control*	1.57	0.85	0.47*	0.03	1	1	1	1	0
Item 5: *loss of interests*	1.64	0.97	0.43*	0.03	1	1	1	1	0
Item 6: *continuation*	1.40	0.84	0.44*	0.04	1	1	1	1	0
Item 7: *deception*	1.45	0.88	0.37*	0.04	1	1	1	1	0
Item 8: *scape*	2.34	1.23	0.38*	0.04	1	1	1	1	0
Item 9: *opportunity loss*	1.21	0.59	0.43*	0.04	1	1	1	1	0
GDT									
Item 1: *impaired control over gaming*	1.76	0.88	0.62**	0.03	1	1	1	1	0
Item 2: *increased priority given to gaming*	1.98	1.03	0.62**	0.03	1	1	1	1	0
Item 3: *continuation despite negative consequences*	1.89	1.05	0.62**	0.03	1	1	1	1	0
Item 4: *experience of significant problems in life*	1.26	0.62	0.63**	0.03	1	1	1	1	0

**Note:** SD = Standard Deviation, *H_j_* = Loevinger’s Coefficient, SE = Standard Error, v. MIIO = Violations of the Manifest Invariant Item Ordering, AISP = Automated Item Selection Procedure, * Medium Discrimination, **Strong Discrimination.

The scalability of the complete scales, measured with Loevinger’s coefficient of homogeneity, was medium for the IGDS9-SF (*H* = 0.45) and strong for the GDT (*H* = 0.62). All item coefficients on both scales exceeded 0.30, suggesting that the items reflect a single latent trait. Specifically, for the IGDS9-SF items, *H_j_* values ranged from 0.51 (item 2) to 0.37 (item 7), including items with low (i.e., items 7 and 8), medium (items 1, 3, 4, 5, 6, and 9), and high (item 2) discrimination values. As for the GDT items, they showed values above 0.62, suggesting strong accuracy, with item 4 being slightly more discriminative than the rest (*H_j_* = 0.63). Furthermore, all the item pair scalability (*H_jk_*) indices for both scales were significantly different from 0 (see Tables S1 and S2 of the Supplementary Material), suggesting that Guttman errors were not detrimental to the overall interpretation of the scores.

The AISP showed that all the items conform to a single scale (AISP = 1) under the four prespecified lower bounds (i.e., 0.05, 0.10, 0.30, 0.55). No violations of the MIIO were found (see Table 2), suggesting that the order of the items was consistent for all respondents, regardless of their total scale score. Furthermore, for both scales, in terms of the degree to which the order of the items remains invariant, the results showed a weak ordering of the items ($H_{IGDS9-SF}^{T}$ = 0.31; $H_{\mathrm{GDT}}^{T}$= 0.41).

It can be concluded that there is a hierarchical order of the items in terms of difficulty. As shown in Table 2, for the IGDS9-SF, the most ‘difficult’ item was item 9 (*negative consequences*; mean score = 1.21), while the ‘easiest’ was item 8 (*escape*; mean score = 2.34). Moreover, for the GDT, the most ‘difficult’ was item 4 (*experience of significant problems in life*; mean score = 1.29), while the ‘easiest’ was item 2 (*increased priority given to gaming*; mean score = 1.98).

Finally, reliability was high for both scales, as the IGDS9-SF showed values of α = 0.84, λ^2^ = 0.85, MS = 0.85, and LCRC = 0.82, while the GDT showed values of α = 0.81, λ^2^ = 0.82, MS = 0.83, and LCRC = 0.82, suggesting a high level of internal consistency.

## 4 Discussion

The present study sought to investigate the psychometric properties of the IGDS9-SF and the GDT using MSA, testing different models (i.e., MHM and DMM). The main findings suggested that both scales were unidimensional, presenting local independence, latent monotonicity, and non-intersecting IRFs. Additionally, both scales obtained high values for the reliability coefficients, further supporting the robustness of reliability of their scores, as shown in previous research (e.g., Evren et al., 2020, Maldonado-Murciano et al., 2020, Maldonado-Murciano et al., 2021, Schivinski et al., 2018, T’ng and Pau, 2020, Yam et al., 2019).

Accordingly, both the IGDS9-SF and the GDT fit the MHM model, suggesting that the sum score of these scales is a robust indicator of disordered gaming levels (Sijtsma & Molenaar, 2002) further allowing: (1) the ordering of the respondents across this latent trait, (2) the use of item scores to assess the difficulty, and (3) observing the hierarchy of the items (Watson et al., 2012). Both scales also fit in the DMM, which signifies that the item difficulty hierarchy remained invariant across all the participants.

In summary, item 2 of the IGDS9-SF (*withdrawal*) and item 4 of the GDT (*experience of significant problems in life*) were the most discriminative items on their respective scales. This finding suggests that these items are particularly effective in differentiating individuals based on the latent trait of disordered gaming. Specifically, higher scores on these items are more strongly associated with higher overall disordered gaming scores, indicating a greater symptom load of disordered gaming.

In terms of difficulty, the items of the IGDS9-SF were ordered from item 9 (*negative consequences*) as the most difficult item to item 8 (*escape*) as the easiest. This finding aligns well with previous IRT studies showing that item 9 requires relatively higher levels of disordered gaming to be endorsed (Gomez et al., 2019, Maldonado-Murciano et al., 2020), and that escapism is highly correlated with disordered gaming (Montag et al., 2019, Montag et al., 2022). Within the APA framework, the potential implications of these findings imply that the experience of negative consequences associated with problematic gaming behavior may be a critical indicator of disordered gaming, therefore, clinicians should pay particular attention when individuals exhibit this symptom, as they may be experiencing a more severe form of disordered gaming. With this information, clinicians may also consider escapism as an early warning sign to identify individuals who may be at risk of developing more severe symptoms of disordered gaming.

From an intervention standpoint, focusing on escapism as an important symptom may be misleading, as video games are often used to escape real-life problems and therefore may not necessarily indicate disordered gaming (Laconi et al., 2017). Furthermore, Stavropoulos et al. (2019) found that individuals with ADHD use video games to escape reality, while Myrseth et al. (2017) concluded that gaming serves as a coping mechanism for depression, loneliness, or an unpleasant reality.

In the GDT, item 4 (*experience of significant problems in life*) presented slightly higher difficulty than the rest of the items. The least ‘difficult’ item was item 2 (*increased priority given to gaming*)*.* This means that within the WHO framework, the experience of significant life problems represents a more severe symptom of disordered gaming. As such, clinicians should pay particular attention to this symptom, as individuals who report experiencing significant life problems due to gaming may be facing a more severe form of the disorder. This symptom may constitute a critical indicator of the severity of disordered gaming, warranting immediate and focused treatment. Furthermore, regarding assigning increased priority to gaming activities, clinicians should consider this symptom as an early warning sign of disordered gaming. Consequently, large-scale interventions in different settings, such as schools, may identify and target individuals who prioritise gaming over other important life activities, as this symptom may aid in early detection and intervention, potentially preventing the escalation of disordered gaming. At the psychometric level, the findings suggest that the items of the GDT may better distinguish between persons with high and low levels of disordered gaming than those of the IGDS9-SF, as the GDT items appear to be slightly more discriminative. This finding is paramount to health practitioners and clinicians when considering which diagnostic framework and tool to use in the assessment of disordered gaming.

Taken together, the present findings add to the growing body of evidence supporting the psychometric properties of the IGDS9-SF and GDT (Beranuy et al., 2020, Jahrami et al., 2024, Karhulahti et al., 2021, Lecuona et al., 2024, Lin et al., 2023, Maldonado-Murciano et al., 2020, Maldonado-Murciano et al., 2021, Sánchez-Iglesias et al., 2020, Wang and Cheng, 2020), further attesting to the fact that both tools are adequate to assess disordered gaming in research and clinical settings.

These findings have important implications for the use of the IGDS9-SF and GDT in preventive interventions and educational settings. For example, in high school settings, counselors and educators can use these scales to identify students who may be at risk of disordered gaming. Early identification can facilitate timely intervention, helping to prevent the escalation of gaming-related issues. For preventive interventions, the scales can be integrated into routine health assessments to monitor individuals’ gaming behaviors. This approach allows for the detection of early warning signs and the implementation of targeted interventions aimed at promoting healthy gaming habits and mitigating potential negative outcomes associated with disordered gaming.

Nevertheless, it is important to consider potential limitations in the present research. The main limitation pertains to the sample selection strategy, as participants were self-selected, making the reported results not generalizable to the Spanish population. Another limitation is the limited representativeness of the sample, as it consists predominantly of young adults and includes a minimum age of 16, excluding younger gamers from the study. Finally, we did not collect information about the type of games the participants play (i.e., online or offline), which also affects the generalizability of the results.

Future studies may help advance the existing literature by further investigating the diagnostic accuracy of the IGDS9-SF and GDT in clinical samples in terms of sensitivity and specificity, and by using a valid and reliable gold standard that allows for the estimation of cut-off points, particularly for the GDT, as this area of research has been explored within the APA framework using the IGDS9-SF (Qin et al., 2020). Another potential line of future research is the validation of both tools across different age ranges, as there is no evidence regarding how they perform in adolescent and elderly populations.

## CRediT authorship contribution statement

**Laura Maldonado-Murciano:** Writing – review & editing, Writing – original draft, Visualization, Validation, Software, Methodology, Investigation, Formal analysis, Data curation, Conceptualization. **Halley M. Pontes:** Writing – review & editing, Writing – original draft, Visualization, Validation, Supervision, Software, Methodology, Formal analysis, Conceptualization. **Maite Barrios:** Writing – review & editing, Resources, Project administration, Methodology, Investigation, Funding acquisition, Data curation. **Juana Gómez-Benito:** Writing – review & editing, Resources, Project administration, Methodology, Investigation, Funding acquisition. **Georgina Guilera:** Writing – review & editing, Writing – original draft, Visualization, Validation, Supervision, Software, Resources, Project administration, Methodology, Investigation, Funding acquisition, Formal analysis, Data curation, Conceptualization.

## Declaration of competing interest

The authors declare the following financial interests/personal relationships which may be considered as potential competing interests: [Laura Maldonado-Murciano, Halley M. Pontes, Maite Barrios, Juana Gómez-Benito, and Georgina Guilera have no conflicts of interest to declare that are relevant to the content of this article].

## Data Availability

Data will be made available on request.
