# Supplementary material for: Mokken scale analysis of the Internet Gaming Disorder Scale–Short-Form and the Gaming Disorder Test
Source: Addict Behav Rep. 2024 Oct 28;20:100567. doi: 10.1016/j.abrep.2024.100567 (PMC11602559; doi:10.1016/j.abrep.2024.100567)
Supplement: Supplementary Data 1 [file mmc1.docx]

**Table S1.** Item-pair scalability indices (H_ijk_) of the Internet Gaming Disorder Scale–Short-Form (IGDS9-SF)

| Items | Item 1 | SE | Item 2 | SE | Item 3 | SE | Item 4 | SE | Item 5 | SE | Item 6 | SE | Item 7 | SE | Item 8 | SE |
| --- | --- | --- | --- | --- | --- | --- | --- | --- | --- | --- | --- | --- | --- | --- | --- | --- |
| Item 1 |  |  |  |  |  |  |  |  |  |  |  |  |  |  |  |  |
| Item 2 | 0.63 | 0.03 |  |  |  |  |  |  |  |  |  |  |  |  |  |  |
| Item 3 | 0.53 | 0.04 | 0.62 | 0.04 |  |  |  |  |  |  |  |  |  |  |  |  |
| Item 4 | 0.54 | 0.04 | 0.56 | 0.04 | 0.57 | -0.04 |  |  |  |  |  |  |  |  |  |  |
| Item 5 | 0.54 | 0.04 | 0.51 | 0.04 | 0.49 | 0.05 | 0.51 | 0.05 |  |  |  |  |  |  |  |  |
| Item 6 | 0.45 | 0.05 | 0.51 | 0.05 | 0.42 | 0.05 | 0.44 | 0.06 | 0.45 | 0.05 |  |  |  |  |  |  |
| Item 7 | 0.40 | 0.05 | 0.41 | 0.05 | 0.30 | 0.06 | 0.39 | 0.05 | 0.31 | 0.06 | 0.42 | 0.06 |  |  |  |  |
| Item 8 | 0.38 | 0.05 | 0.38 | 0.05 | 0.47 | 0.05 | 0.38 | 0.05 | 0.42 | 0.05 | 0.35 | 0.06 | 0.27 | 0.06 |  |  |
| Item 9 | 0.45 | 0.06 | 0.46 | 0.06 | 0.39 | 0.06 | 0.35 | 0.07 | 0.43 | 0.07 | 0.50 | 0.07 | 0.48 | 0.06 | 0.37 | 0.07 |

Note: SE: Standard error.

**Table S2.** Item-pair scalability indices (H_ijk_) of the Gaming Disorder Test (GDT)

| Items | Item 1 | SE | Item 2 | SE | Item 3 | SE |
| --- | --- | --- | --- | --- | --- | --- |
| Item 1 |  |  |  |  |  |  |
| Item 2 | .64 | .03 |  |  |  |  |
| Item 3 | .60 | .03 | .62 | .03 |  |  |
| Item 4 | 63 | .05 | .59 | .05 | .67 | .04 |

Note: SE: Standard error.

**Figure S1.** *Path diagrams of a) the Internet Gaming Disorder Scale–Short-Form (IGDS9-SF) and b) the Gaming Disorder Test (GDT)*

a)

***
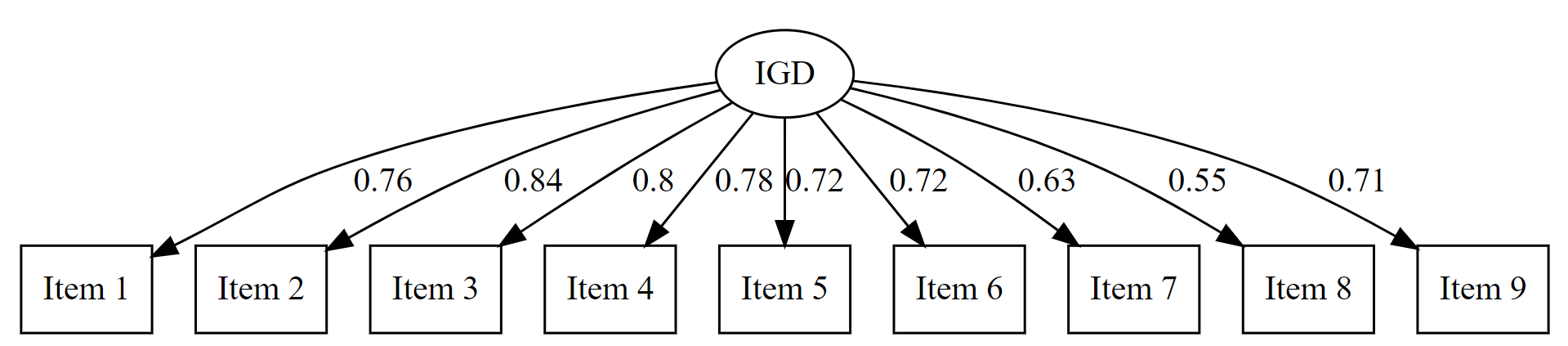
***

b)

**
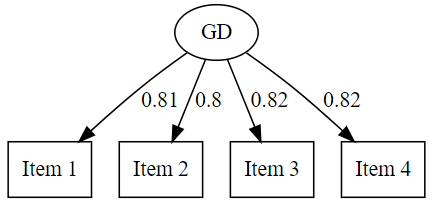
**

**Note:** all coefficients are significant (*p* < .001)

**Figure S2.** *Item Step Response Functions and Item Response Functions of individual items of the Internet Gaming Disorder Scale–Short-Form (IGDS9-SF)*

**
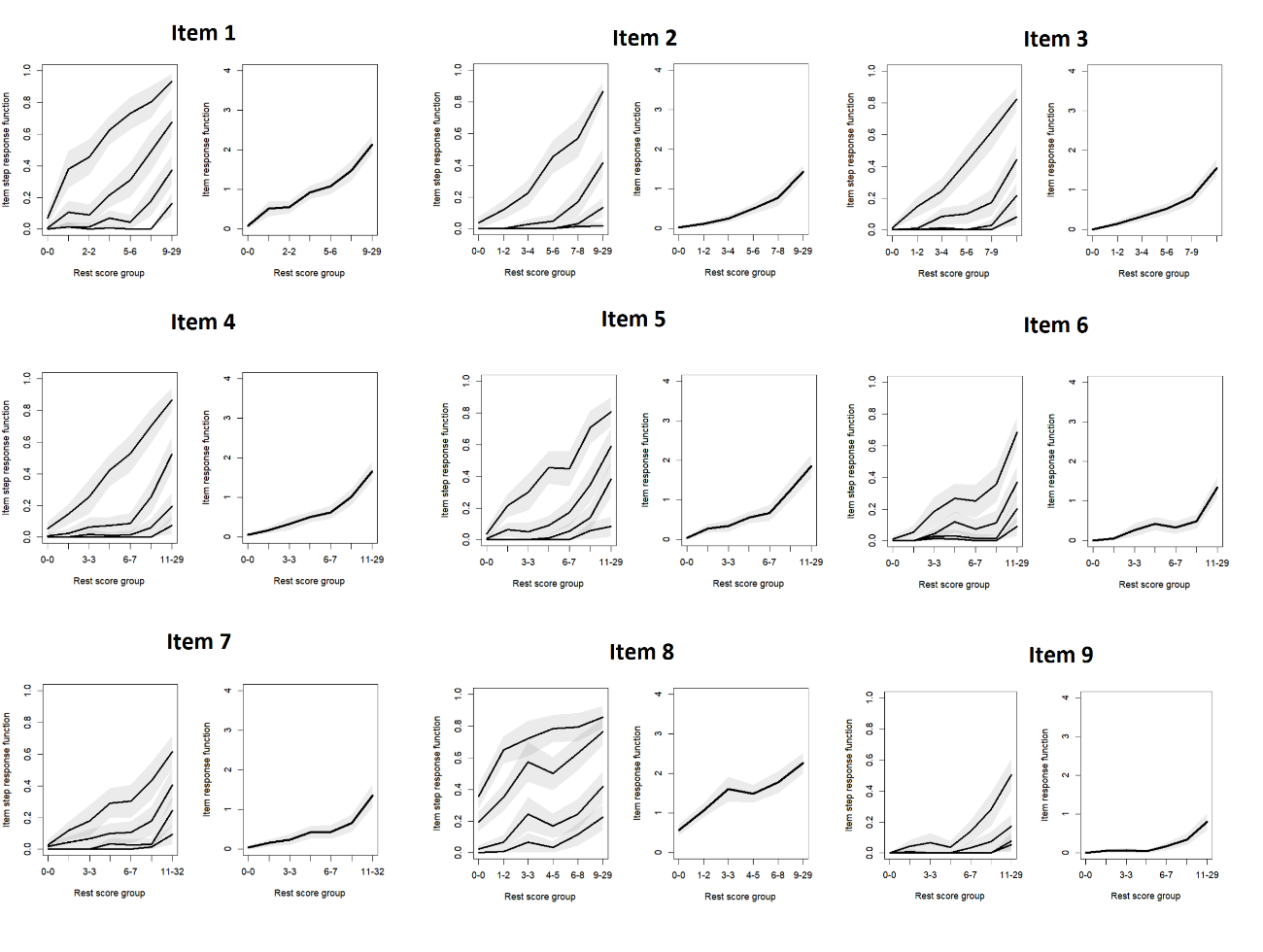
**

**Figure S3.** *Item Step Response Functions* *and* *Item Response Functions of individual items of the Gaming Disorder Test (GDT)***
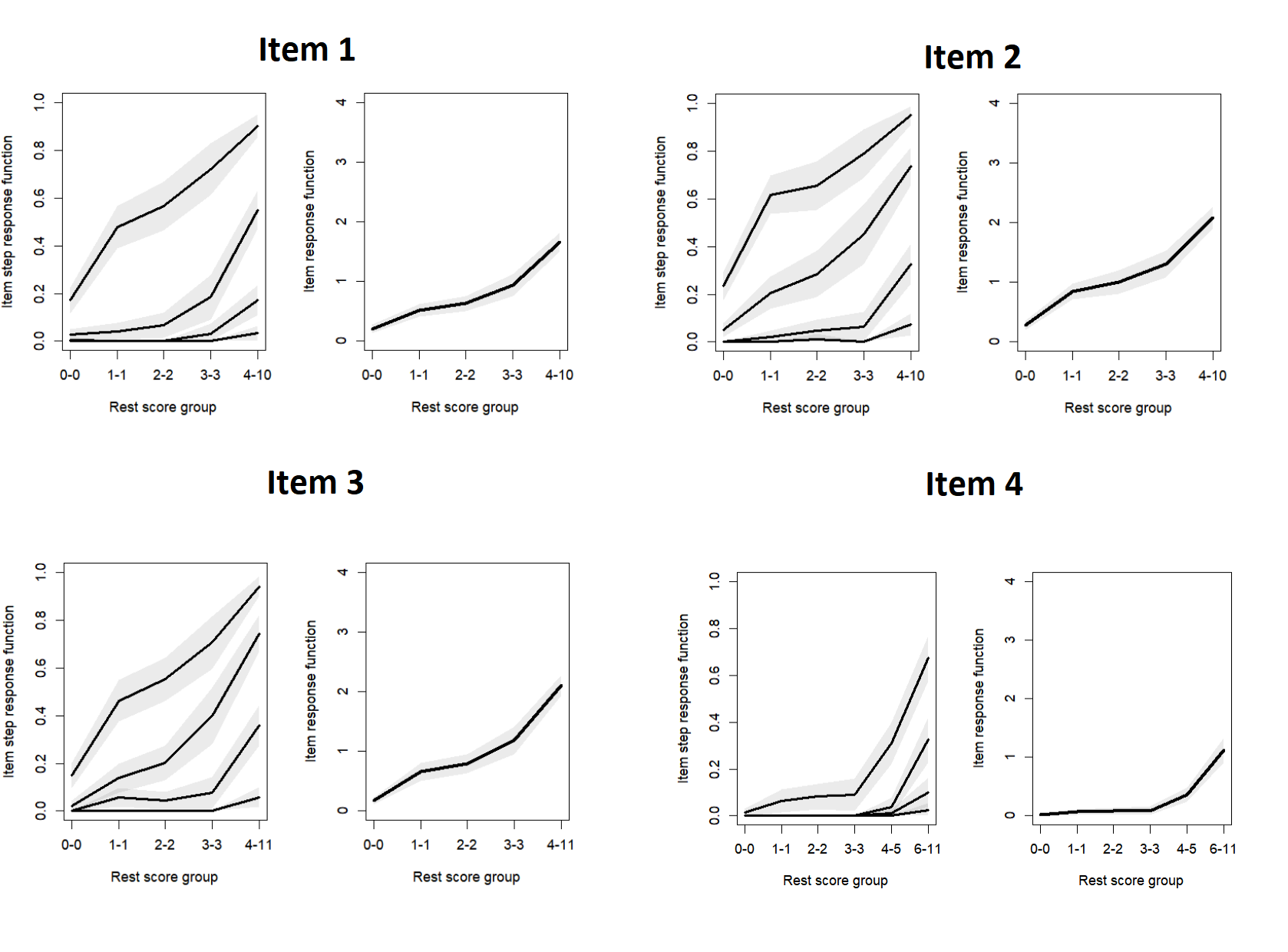
**
